# Endocrine Control of Embryonic Diapause in the Australian Sharpnose Shark *Rhizoprionodon taylori*

**DOI:** 10.1371/journal.pone.0101234

**Published:** 2014-07-03

**Authors:** Daniela Waltrick, Susan M. Jones, Colin A. Simpfendorfer, Cynthia A. Awruch

**Affiliations:** 1 Centre for Sustainable Tropical Fisheries and Aquaculture & School of Earth and Environmental Sciences, James Cook University, Townsville, Australia; 2 School of Zoology, University of Tasmania, Hobart, Australia; 3 CENPAT (Patagonian National Centre)- CONICET, Puerto Madryn, Chubut, Argentina; Baylor College of Medicine, United States of America

## Abstract

The reproductive cycle of the Australian sharpnose shark, *Rhizoprionodon taylori*, includes a temporary suspension of development at the commencement of embryogenesis termed embryonic diapause. This study investigated levels of 17β-estradiol (E_2_), testosterone (T) and progesterone (P_4_) in plasma samples of mature wild female *R. taylori* captured throughout the reproductive cycle and correlated them with internal morphological changes. Levels of T were elevated through most of the embryonic diapause period, suggesting a role of this hormone in the maintenance of this condition. Increasing plasma T concentrations from late diapause to early active development were associated with a possible role of androgens in the termination of embryonic diapause. As in other elasmobranchs, a concomitant increase of E_2_ with ovarian follicle size indicated a direct role of this hormone in regulating vitellogenesis, while a peak in P_4_ suggested this hormone is associated with preovulation and ovulation. Additionally, significant correlations between photoperiod or water temperature and maximum follicular diameter and hepatosomatic index suggest that these abiotic factors may also play a role triggering and regulating the synchrony and timing of reproductive events.

## Introduction

The ability to evolve and adapt reproductive strategies in response to changes in environmental conditions helps ensure the evolutionary success of any species [1]. As such, it has been postulated that environmental seasonality allows for the adjustment of reproductive events to occur over the most favourable period of the year maximizing survival and reproductive success [2]–[4]. By anticipating less favourable conditions, seasonal breeders are able to modify their morphology, physiology and/or behaviour [4]. In order to cope with unfavorable conditions and reduce energy waste, species have evolved several reproductive mechanisms (e.g. delayed fertilization, reduced gestation period), including embryonic diapause [2].

Embryonic diapause is the temporary ceasing or retardation of development during any stage of embryogenesis [5], [6]. The phenomenon has been widely reported for vertebrates and invertebrates. Hypothetically, for live-bearing species delaying gestation allows parturition to occur when the environment is most likely to favour the survival of young [7], [8]. Alternatively, it allows females to restore their energetic reserves after events of ovulation, mating, gestation and birth, without compromising the success of the following reproductive period [9].

In elasmobranchs, embryonic diapause is believed to have evolved independently in at least 16 species displaying three different types of viviparity [9]. This includes species where embryonic development relies solely on yolk (lecithotrophy) or additional nutrient input (matrotrophy) through uterine secretions (aplacental) or directly from the mother (placental) [10]. Aside from the occasional description of embryonic diapause in a number of elasmobranchs, there has been no systematic study of this phenomenon. Investigations have generally been limited to the observation of uterine eggs for extended periods (e.g. [7], [11]). However, the physiological mechanisms controlling this process have never been investigated within this group of vertebrates. Furthermore, the selective pressures that have resulted in the evolution of embryonic diapause, as well as its benefits to the species, remain largely unknown (see review in [9]).

As in other vertebrates, reproductive events in elasmobranchs are associated with periodic cycles of steroid hormones. Therefore, understanding elasmobranch endocrinology is important for accurately delineating reproductive processes [12]–[15]. Testosterone (T), 17β-estradiol (E_2_) and progesterone (P_4_) are the main steroid hormones produced by the ovary [16] and are involved in controlling the reproductive cycle of female elasmobranchs. 17β-estradiol is known to play a role in vitellogenesis [17], [18], while P_4_ can cause an antagonistic effect on E_2_ [19]. Other roles attributed to P_4_ are a regulation of ovulatory events [15], [20], [21], parturition [15], [21], [22], implantation and the inhibition of myometrial contractions [20], [23]. The roles of T are less clear as it shows no distinct patterns among species and therefore it has been associated with several reproductive events (e.g. sperm storage, courtship, ovulation) [20], [24], [25]. Although previous investigations of these three key hormones have led to a better understanding of the physiology of female elasmobranchs, their role in controlling embryonic diapause in this group remains unknown.

The control of embryonic diapause has been better studied in mammals [26] and reptiles [27]. Within viviparous reptiles, low temperature is necessary to arrest the late embryonic development, thus delaying parturition [28]. Although the endocrine control of this trait is not well understood in viviparous reptiles, injections of P_4_ have been shown to delay oviposition [29]. Similarly, concentrations of P_4_ are directly associated with the onset, maintenance and termination of this reproductive trait in mammals [6], in which P_4_ is required to maintain pregnancy and the suppression of its production induces diapause.

Though the control of embryonic diapause seems to be strongly linked to the mother's endocrine functions, previous studies have shown it depends on a complex series of events. An environmental cue, usually photoperiod or temperature, is necessary to trigger the endocrine pathways that regulate the development of the corpora lutea [30], a temporary endocrine gland capable of synthesizing significant amounts of P_4_ [23], [31]. In many cases the administration of P_4_ alone does not induce the termination of diapause. Investigations in mustelids and rodents have shown that a combination of P_4_ with some other unknown luteal protein(s) [32], [33] or estrogen [34], respectively, is necessary to induce the termination of diapause. There is no evidence in the literature for the involvement of T in the control of embryonic diapause in other vertebrate groups.

In this context, the aim of this study was to investigate the role of reproductive hormones, and their correlation with abiotic factors, in the control of diapause and reproductive events in the Australian sharpnose shark *Rhizoprionodon taylori*. This species is a placental viviparous carcharhinid shark endemic to waters across northern Australia and southern Papua New Guinea [35]. *Rhizoprionodon taylori* is an annual reproducer with a gestation period of 11.5 months during which embryonic development is halted at blastodisc stage for a seven-month period (between February and September) of embryonic diapause [8]. This study describes the circulating steroid hormone levels of E_2_, P_4_ and T throughout the female reproductive cycle of *R. taylori* in relation to changes in gonadal morphology and environmental parameters. This is the first time that steroid hormone levels have been described in a diapausing elasmobranch species.

## Materials and Methods

### Sampling

All research activities were conducted under the permit requirements of the Great Barrier Reef Marine Park Authority - GBRMPA (G09/31573.1 and G10/33227.1) and the Queensland Department of Primary Industries and Fisheries – DPI (90911 and 144482). This study was conducted in compliance with the National Health and Medical Research Council (NHMRC) and Queensland Animal Care and Protection Ethics Committee. The protocol was approved by the James Cook University Animal Ethics Committee (permit No. A1508).

Females of *Rhizoprionodon taylori* were collected at night using 10 cm stretched mesh monofilament gillnets in Cleveland Bay, north Queensland (19°14′S, 146°48′E), monthly from February 2010 to February 2012. The nets were checked at 15-minute intervals. Shortly after capture, blood samples (3 ml) were collected from each specimen through caudal venipuncture using pre-heparinised syringes fitted with 22 gauges needles. Blood samples were preserved on ice for no longer than five hours and then centrifuged for five minutes at 1000×g. The separated plasma was stored at -15°C until analysed for hormone levels through radioimmunoassay (RIA).

A total of 152 females were captured. Of these, 29 did not survive capture and were retained for examination of the reproductive organs. Blood samples were collected from the remaining 123 animals, of which 23 were released. All retained animals were euthanised by immersion in benzocaine bath for dissection. Specimens with vitellogenic follicles in the ovary, or embryos or ova in the uterus, were considered mature. Their total stretched length (STL; mm) was measured and total weight (g) and liver weight (g) obtained to calculate the hepatosomatic index (HSI  =  liver mass/body mass × 100). For the analysis of reproductive organs, ovaries were extracted, weighed (g) and the diameter of the largest follicle (MFD) measured to the nearest millimetre; the total number of ova or embryos in each uterus was counted, and total uterus weight (g) recorded, the ova or embryos weighed (g) and measured to the nearest millimetre. Females were classified into five reproductive stages (Table 1).

**Table 1:** Classification of reproductive stages for female *Rhizoprionodon taylori*, where the numbers within the ovary column represent the range of maximum follicular diameter during each reproductive stage.

Stage	Ovary	Uteri
*Post partum*	Vitellogenic follicles (10–14 mm)	Empty
Ovulating	Vitellogenic follicles (∼12.0 mm)	Ova half-way down the oviduct and/or uterus
Diapause	Only previtellogenic follicles (1–3 mm)	Presence of uterine ova
1	Previtellogenic follicles (3–5 mm)	Flaccid uterine ova (not attached to uterus) and visible embryo
2	Previtellogenic and vitellogenic follicles (2.5–13 mm)	Visible embryo attached to the uterus by a placenta

The presence of atretic vitellogenic follicles (AF) and postovulatory follicles (POF) were recorded. Macroscopic and microscopic examinations of ovarian follicles throughout the reproductive cycle were undertaken and correlated to existing literature of the chondrichthyan, especially elasmobranch, ovarian structures. Atresia can also occur in follicles prior to vitellogenesis, however, due to their morphological similarities with POF, only AF were used in the statistical analysis.

Environmental data for the study site were obtained from open online databases. Daily mean water temperature and air pressure data were obtained from the Australian Institute of Marine Science [36], and data on day length throughout the study period were obtained from U.S. Naval Observatory Astronomical Applications Department [37].

### Radioimmunoassay

Plasma levels of P_4_, T and E_2_ were determined by RIA in 123 plasma samples. Plasma aliquots of 100 µl were extracted with ethyl acetate (1 ml) and 100 µl aliquots were then analysed. The antiserum and [1,2,6,7-^3^H] for E_2_ and P_4_ were purchased from Sigma-Aldrich (Australia). The E_2_ and P_4_ antiserum were reconstituted by adding 5 ml of Tris buffer (pH 8, 0.1 m HCl) and 50 µl of [1,2,6,7-^3^H] E_2_ and P_4_ were diluted in 5 ml of 100% ethanol and kept as stock for the assay. Duplicate standards (0–800 pg/tube authentic E_2_ and P_4_ in ethanol) and sample extracts were dried down and 200 µl of the reconstituted antiserum, diluted 1∶10 in assay buffer (containing 0.1% of gelatin and 0.01% of Thimerosal), and 100 ul of the E_2_ and P_4_ stock, diluted 1∶9 in assay buffer, added to each tube. Samples were placed in a bath at 37°C for an hour. Bound and free fractions were then separated using dextran-coated charcoal and aliquots of the supernatants counted in a Beckman LS 5801 liquid scintillation counter. All assays were validated by the evaluation of the slope of serially diluted extractions of plasma against the assay standards. Extraction efficiency was determined from recovery of ^3^H–labelled steroid added to pooled aliquots of plasma and assay values corrected accordingly. Extraction efficiency was 86% (E_2_) and 90% (P_4_). The detection limit for all assays was 0.02 ng.ml^−1^. Intra and interassay variability was determined by including in each assay replicates of three levels of commercially available human control serum (CON 4, CON5, and CON 6 DPC). Interassay variability was 7% (E_2_) and 9% (P_4_) and intrassay variability less than 5%.

The T antiserum was Sirosera C-6050 (Bioquest) and [1,2,6,7-^3^H] T was purchased from Sigma-Aldrich (Australia). Duplicate standards (0–800 pg/tube authentic testosterone in ethanol) and sample extracts were dried down and the assay protocol used was as described by Nicol *et al.* [38]. The assay was validated by the evaluation of the slope of serially diluted extractions of plasma against the assay standards. The detection limit for the assay was 0.02 ng.ml^−1^. Extraction efficiency was 90%, interassay coefficient of variation was 11% and intrassay variability was less than 4%.

### Statistics

All statistical analyses were conducted using R (version 2.15.0) [39] with a critical probability level of 0.05. Hormone concentrations failed the Kolmogorov-Smirnov test for normality and therefore nonparametric statistic tests were used. Differences in sample medians (steroid hormone, STL and HSI) over time and different stages were evaluated by Kruskal-Wallis one-way ANOVA on ranks followed by a pairwise Wilcoxon rank sum tests with Bonferroni adjustment. The Spearman correlation [40] was used to determine the existence of associations between hormone levels, environmental parameters and morphometric parameters.

## Results

### Reproductive cycle

Females ranging from 517 to 975 mm STL (688±4 mm; mean ±SE) were captured in all months of the year, except in June. Mean STL did not differ significantly between months (Kruskal Wallis, rho  = 15.48, df = 10, p = 0.12).

**Figure 1:**
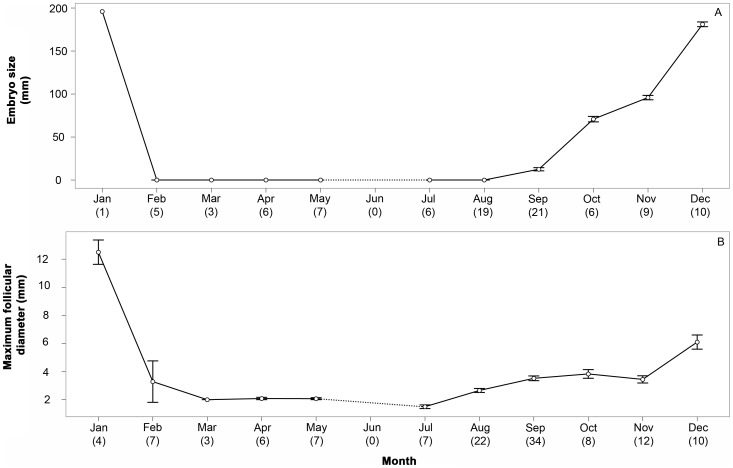
Average monthly size (±SE) of (A) embryos and (B) maximum ovarian follicles in *Rhizoprionodon taylori*. Numbers in brackets denote sample size.

The average litter size per female was 5.3±0.2 embryos (range: 2–10). Soon after parturition (January), diapausing ova consisting of bright-yellow dense yolk masses concealed in brown egg cases measuring 23.9±0.3 mm (range: 21–29 mm) were observed in the uterus from mid February to mid September. Upon termination of diapause (stage 1), uterine ova became flaccid and slightly increased in size (29±0.2 mm, range: 25–32 mm). At this stage in September embryos from 15 to 17 mm were first visible. Embryos grew continuously to ∼212 mm in January, when parturition occurs (Figure 1A).

The development of ovarian follicles commenced in late diapause (August). This initially slow process sped up in the two months prior to parturition (December to January; Figure 1B), when the largest embryonic size was recorded. Independent of the litter size, a single AF was present in the ovary throughout the diapausing period. The maximum size of these structures (26 mm) was observed in postovulatory females, after which it continuously reduced in size. The identification of AF at the end of the diapausing period (August – September) was difficult and sometimes not possible due to its small size, similar to POF (1–2 mm).

**Figure 2:**
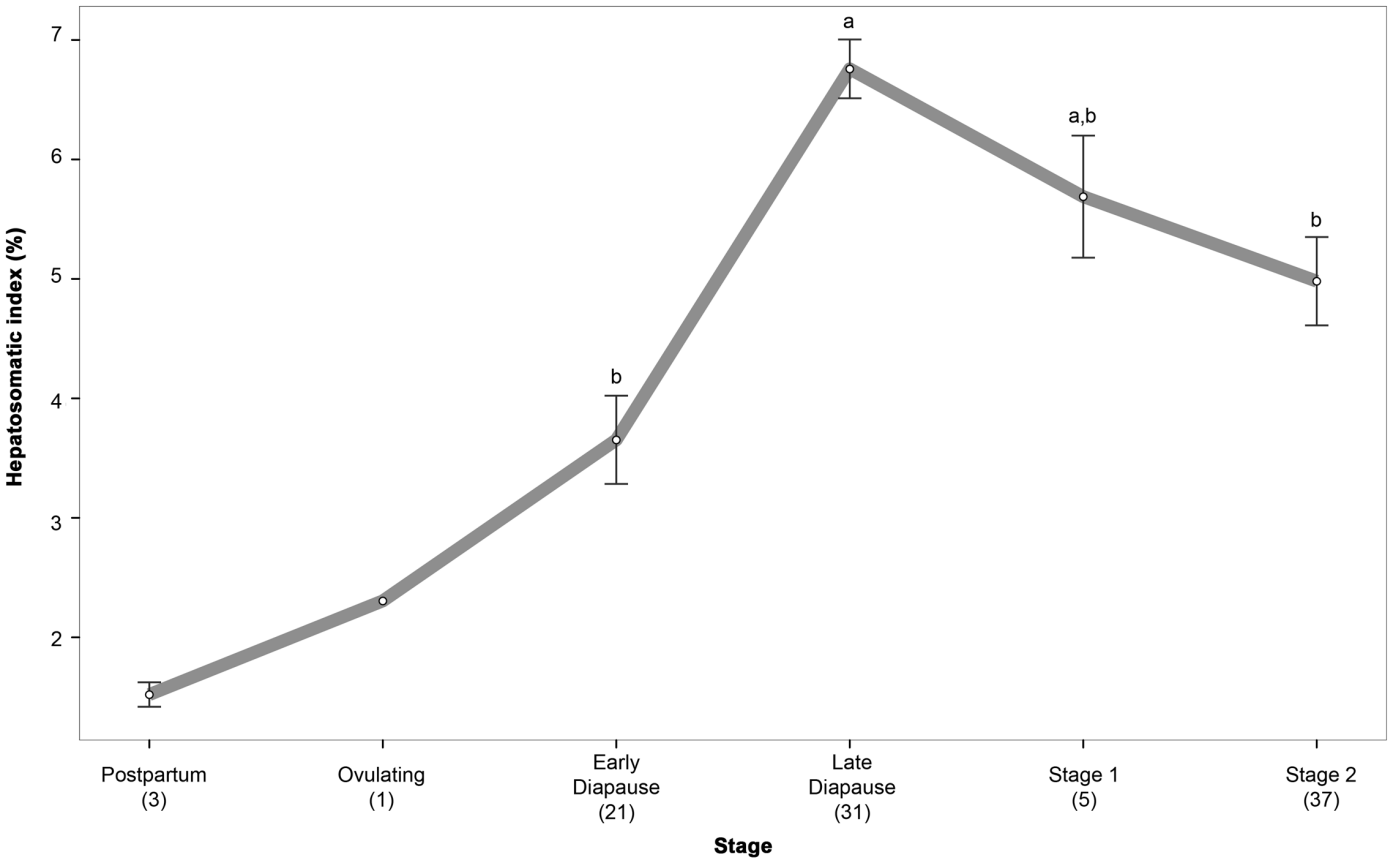
Hepatosomatic index variation throughout reproductive cycle in female *Rhizoprionodon taylori*. Values are medians ±SE. Letters depict statistical similarity using pairwise Wilcox post-hoc test. Missing letters represent groups that are too small for statistical analysis. Numbers in brackets denote sample size.

The HSI was significantly different between stages throughout the reproductive cycle, ranging from 1.2% in *post partum* females, to 9.6% in late diapause females (χ^2^ [5]  = 36.341, p<0.001) (Figure 2).

### Serum steroid analysis

**Figure 3:**
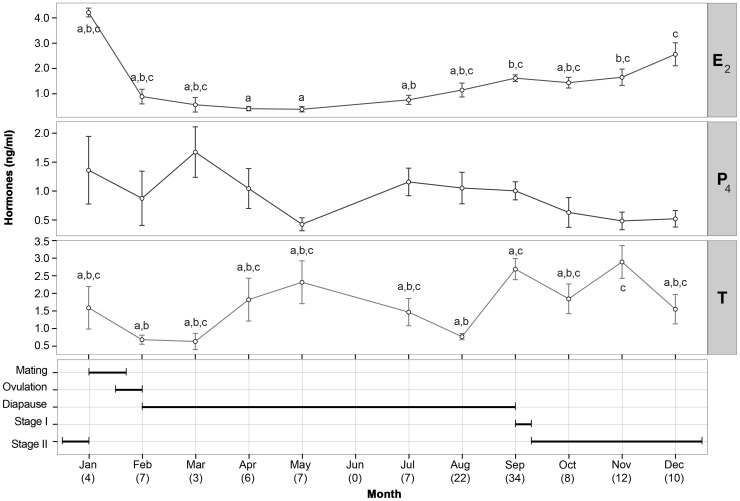
Seasonal variation of 17β-estradiol (E_2_), progesterone (P_4_) and testosterone (T) in female *Rhizoprionodon taylori*. Letters depict statistical similarity using pairwise Wilcox post-hoc test. Progesterone levels were not significantly different throughout the year. The graph at the base of the figure indicates the timing of each reproductive event in females and numbers in brackets denote sample size.

All reproductive hormones showed distinct cycles throughout the year (Figure 3) and no significant correlation was observed among them (P_4_ vs. E_2_: r_s_[123]  = −0.110, p = 0.23; P_4_ vs. T: r_s_[123]  = −0.011, p = 0.90; E_2_ vs. T: r_s_[123]  = 0.150, p = 0.10). Restricted sample sizes of different stages in January and February did not allow statistical determination of median differences amongst steroids at different stages over this period. However, differences in hormone levels between stages at each given month were examined when necessary.

### 17β-Estradiol

**Figure 4:**
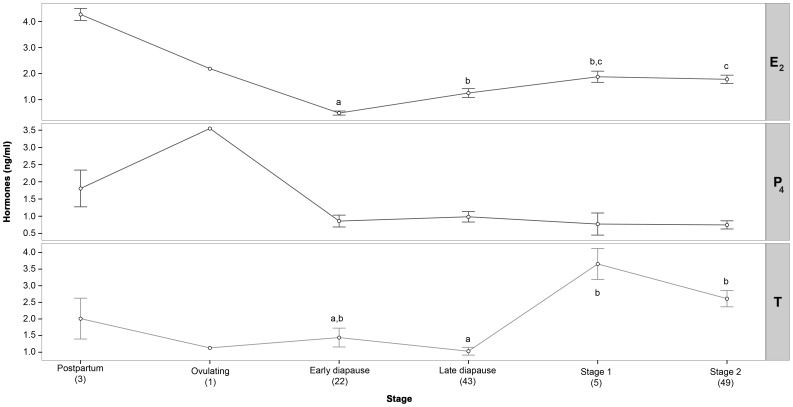
Mean plasma steroid hormone concentrations at different reproductive stages of *females Rhizoprionodon taylori*. Letters depict statistical dissimilarity using pairwise Wilcox post-hoc test. Missing letters in E_2_ and T graphs represent groups with sample sizes too small for statistical analysis. Progesterone levels were not significantly different among reproductive stages. Numbers in brackets denote sample size.

Plasma of E_2_ (1.42±0.13; range: 0.03–6.2 ng.ml^−1^) varied significantly between stages through the reproductive cycle. Concentrations remained low through most of the diapausing period, starting to increase at the beginning of follicular development. Levels gradually increased to a peak at late stage 2 and *post partum* females before declining prior to ovulation (Figure 4). A Kruskal Wallis test revealed significant differences between E_2_ levels at different reproductive stages (χ^2^ [5]  = 46.52, p<0.01), with significant differences between levels of this hormone in early diapause and late diapauses (stage 2 and stage 3, respectively).

**Figure 5:**
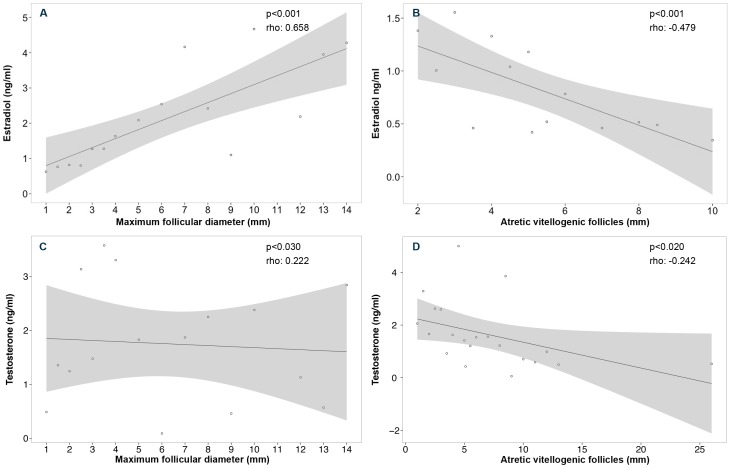
Relationship between plasma levels of hormones and ovarian follicles in *Rhizoprionodon taylori*. Changes in 17β-estradiol were significantly correlated with (A) maximum follicular diameter, and (B) atretic vitellogenic follicles observed from February to October. A weak correlation was observed between testosterone and (C) maximum follicular diameter, and (D) atretic vitellogenic follicles observed from February to October. Graphs show the correlation, including regression line and confidence interval of 95% represented by the shaded area.

17β-estradiol levels were significantly correlated to ovarian structures. This association was positive with MFD (r_s_[100]  = 0.658, p<0.001; Figure 5A) and negative when correlated with the size of AF observed during embryonic diapause, from February to October (r_s_ [54]  = −0.479, p<0.001; Figure 5B). Hence, it appears that E_2_ may be associated with the control of ovarian functions.

### Progesterone

Serum levels of P_4_ (0.900±0.082; range: 0.02–5.49 ng.ml^−1^) in female *R. taylori* remained relatively low and were not significantly different between months (Figure 3). However, animals at different reproductive stages in January and February showed distinct differences in P_4_ concentration. The only stage 2 female captured in January showed a P_4_ level that was almost undetectable (0.02 ng.ml^−1^), being much lower than the three *post partum* females (1.81±0.53, range: 0.97–2.80 ng.ml^−1^). Progesterone levels then increased in February to a peak (3.55 ng.ml^−1^) in one ovulating female before a sharp decrease observed in six females in early diapause (0.43 ng.ml^−1^±0.17, range: 0.02–0.85 ng.ml^−1^). Although there was no significant correlation of this hormone with the female's reproductive stage (χ^2^ [5] = 9.329, p = 0.10), MFD (r_s_[100]  = −0.014, p = 0.89) or AF size (r_s_[95]  = 0.147, p = 0.15), P_4_ levels showed a trend increasing from late pregnancy to a peak at ovulation before returning to basal levels at early gestation (Figure 4).

### Testosterone

Plasma T levels (1.86±0.17, range: 0.02–7.20 ng.ml^−1^) varied widely throughout the year (Figure 3). In January and February, where sample sizes of different stages were too low to allow statistical comparisons some distinct variations were observed. In January, a rapid increase in T level was observed from one stage 2 female (0.33 ng.ml^−1^) compared to three *post partum* females (2.01±0.62; range: 0.81–2.84 ng.ml^−1^), before returning to basal levels in February at ovulation and early diapause (0.68±0.13; range: 0.06–1.13 ng.ml^−1^). Therefore, it appears that T levels may undergo a small ephemeral *post partum* peak before declining at ovulation.

Testosterone varied significantly throughout different reproductive stages (χ^2^ [5]  = 29.87, p<0.01; Figure 4). Levels of this hormone were significantly different between late diapause and stages 2 and 3, nevertheless, it is important to note that high temporal variability of T concentrations during each given reproductive stage influenced this result. In fact, T concentrations remained elevated through most of the embryonic diapause period, after which two distinct peaks were observed (Figure 3). The first one, in September, revealed a significant difference in plasma T levels at different stages occurring in September (χ^2^ [2]  = 13.9, p<0.01). Significant differences in T in late diapausing females (1.09±0.20; range: 0.39–2.93 ng.ml^−1^, n = 11) compared to those at active development stages 1 and 2 (3.46±0.33; range: 0.53–7.09 ng.ml^−1^, n = 23) were observed (Figure 4). This result suggests a possible role for T in the termination of diapause. The second peak occurred at Stage 2, in November, and was not correlated to any observed morphological change. Testosterone levels showed a weak positive correlation to MFD (r_s_[100]  = 0.22, p = 0.027; Figure 5C) and negative to AF size (r_s_[95]  = −0.24, p = 0.018; Figure 5D), thus indicating that to some extent this hormone may be required in the regulation of ovarian functions.

### Abiotic factors and reproduction

**Figure 6:**
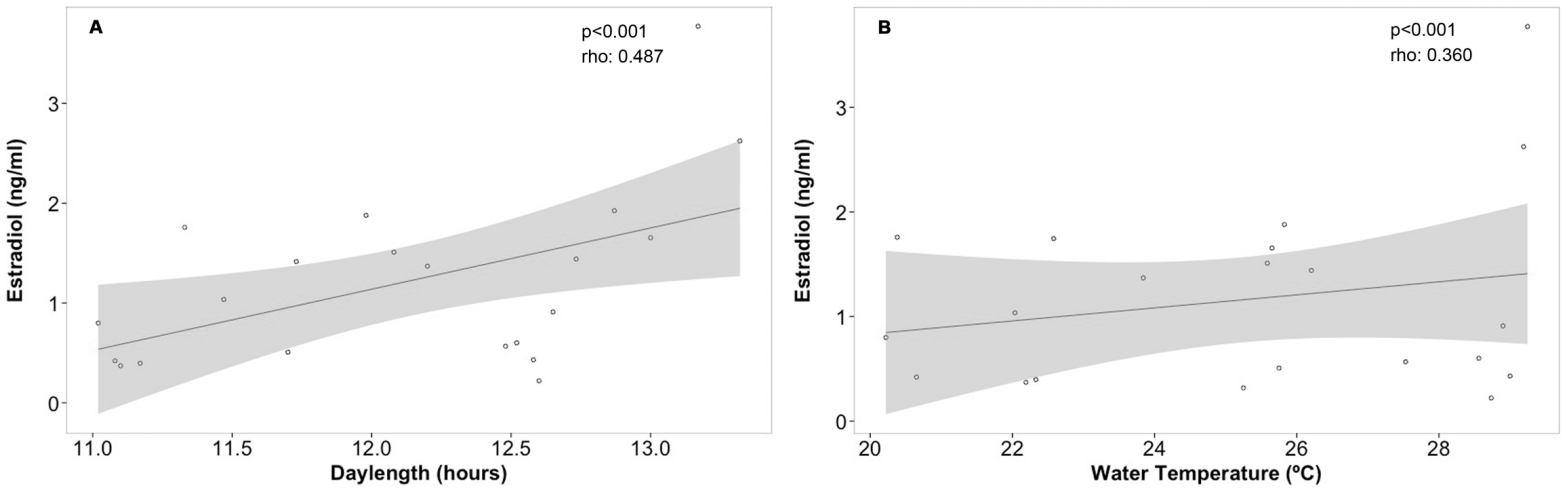
Seasonal changes in 17β-estradiol levels in *Rhizoprionodon taylori* were positively correlated with changes in environmental parameters: (A) day length and (B) water temperature. Graphs show the correlation, including regression line and confidence interval of 95% represented by the shaded area.

The water temperature in Cleveland Bay varied in close association with day length. A maximum seawater temperature of 30.1°C was recorded in February 2010 and January 2011 and after this peak, the temperature dropped gradually to a minimum of 20.8°C and 19.6°C during winter in July 2010 and June 2011 respectively. Day length at the study site latitude varied from 13.3 h in December to 10.9 h in June. Statistical analysis showed significant correlation between the environmental variables and E_2_ (day length: r_s_[123]  = 0.487, p<0.001, Figure 6A; water temperature: r_s_[127]  = 0.360, p<0.001, Figure 6B), but not with T or P_4_. Among the measured biological parameters, environmental parameters have also shown a significant correlation with HSI (day length: r_s_[98]  = −0.557, p<0.001; and water temperature: r_s_[96]  = −0.664 p<0.001) and MFD (day length: r_s_[100]  = 0.656, p<0.001; and water temperature: r_s_[98]  = 0.559, p<0.001).

## Discussion

This is the first assessment of hormone levels of a diapausing elasmobranch. It provides a quantitative description of the main circulating reproductive hormones throughout the annual reproductive cycle. The amplitude of hormonal change in female *R. taylori* in association with reproductive events suggests some involvement of E_2_, T and P_4_ in the regulation of these events. Unlike mammals and reptiles, T and P_4_ (to a lesser degree) are probably involved in the control of embryonic diapause, but only T seems to be required at the release from embryonic diapause and reactivation of active development. Although E_2_ does not appear to have a direct role in controlling diapause, evidence suggests that photoperiod influences levels of this hormone, which could possibly be involved in the regulation and synchronization of reproductive events.

### Late pregnancy and ovulation events

Although only small sample sizes were obtained from animals at the two stages occurring in both January (late stage 2 and *post partum*) and February (ovulating and diapause), the patterns of hormonal change were similar to previously studied elasmobranchs, allowing some assumptions to be made. Preovulatory peaks of E_2_ and T levels followed by increments in P_4_ concentrations at ovulation (when E_2_ and T levels sharply drop), observed in *R. taylori*, have been previously reported in a number of oviparous (*Raja erinacea*: [41], [42]) and viviparous elasmobranchs (*Negaprion brevirostris*: [22]; *S. tiburo*: [20]; *S. acanthias*: [41]). A surge of T during preovulatory events is commonly linked with reproductive behaviour, ovulation and courtship events [15], [20]–[22], while a peak in P_4_ is associated with the termination of the vitellogenic process. Therefore, it seems that in *R. taylori*, increased P_4_ and T levels are necessary to induce ovulation and inhibit further enlargement of vitellogenic follicles stimulated by E_2_.

The liver is known to be an important energetic reservoir, utilized in the development and maturation of large follicles and the nourishment of the developing embryos [43], [44]. Commonly, mating and fertilization quickly follow parturition in annual breeders with concurrent ovarian and gestation cycles, such as *R. taylori* [8], [45]. Nonetheless, all seasonally breeding elasmobranchs with consecutive ovarian cycles and gestation periods (*Carcharhinus isodon*: [46]; *Carcharhinus plumbeus*: [47]) require a ‘resting stage’, usually one year, to recover their hepatic reserves and produce large vitellogenic follicles in preparation for the following gestation [45], [46].

This study demonstrates that levels of HSI in *R. taylori* steadily increased from minimum, at the early stages of pregnancy and diapause, to maximum at late diapause. Therefore, it seems that a diapausing period, as the resting stage, could allow females to restore energetic reserves in preparation for the subsequent ovarian cycle and embryonic nourishment, once development is restarted. Although more studies are required to confirm this theory, it seems that embryonic diapause allows females to restore their fitness between gestations without undergoing a biennial cycle.

### Maintenance of diapause

Moderate levels of P_4_ in *R. taylori* throughout most of the diapausing period (except in May) indicate a possible role of this steroid in maintaining this phase of arrested development. Progesterone is closely related with vertebrate gestation and is generally required to allow embryonic development of mammals. In fact, where diapause occurs within this group, P_4_ production is inhibited during the period of arrested development, only allowing enough secretion to maintain the viability of the embryos [5], [48]. However, this steroid hormone seems to have evolved different roles among elasmobranchs, as only a few species have been shown to elevate P_4_ levels through the gestation period (e.g. *Sphyrna tiburo*: [20]; *Squalus acanthias*: [49]). The role of P_4_ in *R. taylori* seems to be diametrically opposed to its role in mammals playing a role more related with the maintenance than the inhibition of diapause.

Nevertheless, it is also possible that P_4_ does not play a direct role in the inhibition of active embryonic development. Another role attributed to P_4_ in elasmobranchs is the inhibition of spontaneous uterine contractions, maintaining a quiescent uterus for recently formed embryos in *S. acanthias* [50]. Sorbera and Callard [50] suggest that uterine contractions allow the flushing of seawater into the uterine cavity, providing oxygenated water and the removal of waste materials. Assuming that, as in other vertebrates, diapausing embryos of *R. taylori* have very low metabolic rates [6], [51], the exchange of water and removal of waste material is unnecessary and could potentially damage the delicate embryos. Therefore it seems possible that, as in *S. acanthias*, sustained moderate P_4_ levels in diapausing *R. taylori* could play a role in inhibiting uterine contractions, providing embryos with a quiescent uterus through this period.

The major site of P_4_ synthesis in elasmobranchs has been postulated to be the CL, which can be formed after ovulation from the remnants of POF or from AF [52]. Histochemical studies have identified luteinizing tissues in POF [53] and AF [54], or both [55], in several elasmobranch species. The presence of both POF and AF persisting in the ovary through most of the diapausing period suggests these structures could be responsible for the observed sustained P_4_ levels during embryonic diapause. The lack of a significant correlation between AF size and levels of P_4_ in *R. taylori* could indicate that the synthesis capacity of this temporary gland is not directly associated with its size. Alternatively, this possibly indicates that the POF or other structures could potentially be more important for P_4_ production in this species. Studies in reptiles have demonstrated the capacity of P_4_ synthesis within the placental and ovarian tissues [56], [57]), suggesting that in *R. taylori* other sites of P_4_ production may exist.

Elevated T concentrations through most of the diapausing period (April to July) suggest this hormone may also function in the maintenance of embryonic diapause. Androgens have never been reported to have an involvement in the regulation of diapause of mammals or reptiles. Within non-diapausing elasmobranchs, concentrations of T are generally low during pregnancy and do not seem to have a specific role during this period. The exception is *Dasyatis sabina* [21], where T was associated with changes in uterine events (embryonic nourishment). Histotroph secretion (uterine nourishing secretions) in *R. taylori* only starts at the release from embryonic diapause [58], therefore increased T levels during this period could suggest this androgen is required to maintain embryo viability while in the arrested development period.

Contrary to P_4_ and T, which seem to be associated with the regulation of some reproductive events, including embryonic diapause, the major role of E_2_ appears to be follicular growth and maturation. The present data demonstrates a strong linkage between increasing levels of E_2_ and vitellogenesis, which increase concomitantly from late diapause to a maximum in *post partum* animals. Similar patterns have been reported for several elasmobranchs studied to date [15], [21], [23], [24], [41], corroborating earlier *in vitro* studies where the E_2_ synthesis capacity by the elasmobranch ovarian follicles has been demonstrated (Fileti and Callard, 1990 apud [59], [60]). Once synthesised and secreted, E_2_ has been shown to induce the liver to produce vitellogenin, the main yolk precursor [16], [41]. Thus, the strong correlation between E_2_ levels and MFD observed in this study reflects this vitellogenin production by the liver [41], [43]. Therefore, it seems that this sex steroid is not directly involved in the regulation of embryonic diapause but, as in other elasmobranchs, it is essential for the control of vitellogenesis of *R. taylori*.

### Termination of diapause and active development

Among the measured hormones, the termination of embryonic diapause and reactivation of embryonic development in *R. taylori* possibly only requires the presence of T. Unlike previous hypotheses that, as in other diapausing vertebrates, P_4_ would play an important role at the reactivation of normal development in elasmobranchs [9], the data in the present study do not show a peak in P_4_ prior to or at the release from embryonic diapause in September. Instead, a 3-fold increase in levels of T is observed in females from late diapause to stages 1 and 2, suggesting this hormone is possibly required for the reactivation of embryonic development and therefore terminating diapause. In addition, the new ovarian cycle starts as diapause is restarted, so ovarian follicles are very small at early embryonic stages until large (ready to ovulate) at parturition, supporting the idea that T may play a role in the reactivation of embryo development than triggering the initial vitellogenesis stages. Although the role that T plays in viviparous elasmobranch remains unclear, T has been associated with the later stages of follicle growth in oviparous elasmobranchs [42], [61], [62]. However, further studies are required to confirm whether these levels are specifically associated with embryonic development or other reproductive process, such as follicular development.

A further decline in P_4_ levels following the reactivation of active development supports the suggestion that this sex steroid could be playing an inhibitory role in embryonic development and/or regulation of uterine contractions. Testosterone, on the other hand, remains moderately elevated through the remaining part of gestation. A peak in November is not associated with any change in morphometric parameters, however previous observations by Simpfendorfer [58] suggest that the timing could be associated with the transition of embryonic nourishment (from histotroph to placenta). Testosterone has been previously associated with changes in embryonic nutrition in at least one elasmobranch. Embryos of *D. sabina* are nourished by yolk followed by uterine secretions and this transition is associated with a peak in T [21]. Thus, T could possibly play a role in the uterine events related to embryonic nutrition.

### Environmental factors

The present data indicates that abiotic factors may play a role as reproductive cues to synchronize reproductive events in *R. taylori*. Strong relationships were shown between the abiotic factors and MFD and HSI, suggesting that a certain level of dependency could exist between these variables and the changes in environmental factors. Additionally, the correlation of abiotic factors and E_2_ supports the hypothesis that activating the synthesis of this hormone in this species possibly depends on environmental conditions.

Although the use of external factors as cues to regulate the timing of reproductive cycle is well known in other vertebrates [3], [26], this strategy is not well documented in male and female elasmobranchs. While an association between abiotic factors and hormone levels has been demonstrated in at least three elasmobranchs (*Hemiscyllium ocellatum*: [63]; *Urobatis halleri*: [64], [65]; *Scyliorhinus canicula*: [61], controlled experiments demonstrating such dependence has only been shown in male *U. halleri* [64], in which temperature has a role in elevating T levels.

Whether photoperiod or water temperature acts as reproductive cues controlling reproductive events (including embryonic diapause) in *R. taylori* and other elasmobranchs requires further investigation in controlled environments. Thus, it seems likely that highly synchronous populations, such as *R. taylori* in Cleveland Bay, require a fairly stable element to allow changes in reproductive stages to occur a short period apart among individuals.

## Conclusions

This study provides the profile of hormones throughout the reproductive cycle of a diapausing elasmobranch and suggests that *R. taylori* may have evolved distinct endocrine pathways from other studied diapausing vertebrates. The data in the present study suggest the involvement of P_4_ and T in the control of embryonic development, while only T appears to be required for the reactivation of embryonic development after diapause. However, further research will be necessary to confirm the presence of androgen targets on the female and embryo, as well as the effects of this androgen in embryonic development. As in other elasmobranchs, levels of E_2_ in this species are closely associated with follicle development. This investigation provides support for the regulation of reproductive events by environmental variables. It provides indication that environmental factors such as day length and temperature may be playing a role in triggering events such as vitellogenesis and embryonic diapause in *R. taylori*.

## References

[1] Norris DO (2007) The endocrinology of mammalian reproduction. Vertebrate endocrinology. 4 ed. San Diego, CA: Elsevier Academic Press. pp. 322–370.

[2] Angelini F, Ghiara G (1984) Reproductive modes and strategies in vertebrate evolution. Bolletino di zoologia 51: 121–203.

[3] Hastings M, Herbert J, Martensz N, Roberts A (2006) Annual reproductive rhythms in mammals: mechanisms of light synchronization. Annals of the New York Academy of Sciences 453: 182–204. 10.1111/j.1749-6632.1985.tb11810.x 2934016

[4] Jacobs JD, Wingfield JC (2000) Endocrine control of life-cycle stages: a constraint on response to the environment? The Condor 102: 35–51.

[5] Mead RA (1993) Embryonic diapause in vertebrates. The Journal of Experimental Zoology 266: 629–641. 8371102 10.1002/jez.1402660611

[6] Renfree MB, Shaw G (2000) Diapause. Annual Review of Physiology 62: 353–375. 10.1146/annurev.physiol.62.1.353 10845095

[7] Marshall LJ, White WT, Potter IC (2007) Reproductive biology and diet of the southern fiddler ray, *Trygonorrhina fasciata* (Batoidea: Rhinobatidae), an important trawl bycatch species. Australian Journal of Marine and Freshwater Research 58: 104–115.

[8] Simpfendorfer CA (1992) Reproductive strategy of the Australian sharpnose shark, *Rhizoprionodon taylori* (Elasmobranchii: Carcharhinidae), from Cleveland Bay, Northern Queensland. Australian Journal of Marine and Freshwater Research 43: 67–75.

[9] Waltrick D, Awruch C, Simpfendorfer C (2012) Embryonic diapause in the elasmobranchs. Reviews in Fish Biology and Fisheries 22: 849–859.

[10] Musick JA, Ellis JK (2005) Reproductive evolution of Chondrichthyans. In: Hamlett WC, editor. Reproductive biology and phylogeny of Chondrichthyes: sharks, batoids and chimaeras. Enfield, New Hampshire: Science publishers, INC. pp. 45–79.

[11] Yamaguchi A. Reproductive biology of Longheaded Eagle Ray, *Aetobatus flagellum*, in Ariake Bay, Kyushu, Japan. In: Donnelly MA, Cooper KE, editors; 2006; New Orleans.

[12] Awruch CA, Frusher SD, Pankhurst NW, Stevens JD (2008) Non-lethal assessment of reproductive characteristics for management and conservation of sharks. Marine Ecology Progress Series 355: 277–285.

[13] Callard IP, George JS, Koob TJ (2005) Endocrine control of the female reproductive tract. In: Hamlett WC, editor. Reproductive biology and phylogeny of Chondrichthyes sharks, batoids and chimaeras.Plymouth, UK: Sciences Publishers Inc. pp. 283–300.

[14] Sulikowski J, Tsang P, Howell W (2005) Age and size at sexual maturity for the winter skate, *Leucoraja ocellata*, in the western Gulf of Maine based on morphological, histological and steroid hormone analyses. Environmental Biology of Fishes 72: 429–441.

[15] Tricas TC, Maruska KP, Rasmussen LEL (2000) Annual Cycles of Steroid Hormone Production, Gonad Development, and Reproductive Behavior in the Atlantic Stingray. General and Comparative Endocrinology 118: 209–225. 10890563 10.1006/gcen.2000.7466

[16] Gelsleichter J (2004) Hormonal regulation of elasmobranch physiology. In: Carrier JC, Musick JA, Heithaus MR, editors. Biology of sharks and their relatives.Boca Raton: CRC press. pp. 287–323.

[17] Callard IP, Etheridge K, Giannoukos G, Lamb T, Perez L (1991) The role of steroids in reproduction in female elasmobrachs and reptiles. Journal of Steroid Biochemistry and Molecular Biology 40: 571–575. 1958559 10.1016/0960-0760(91)90278-d

[18] Craik JCA (1978) The effects of oestrogen treatment on certain plasma constituents associated with vitellogenesis in the elasmobranch *Scyliorhinus canicula* L. General and comparative Endocrinology 35: 455–464. 720816 10.1016/0016-6480(78)90141-7

[19] Perez LE, Callard IP (1993) Regulation of hepatic vitellogenin synthesis in the little skate (*Raja erinacea*): Use of a homologous enzyme-linked immunosorbent assay. Journal of Experimental Zoology 266: 31–39.

[20] Manire CA, Rasmussen LEL, Hess DL, Hueter RE (1995) Serum Steroid Hormones and the Reproductive Cycle of the Female Bonnethead Shark, *Sphyrna tiburo* . General and Comparative Endocrinology 97: 366–376. 7789751 10.1006/gcen.1995.1036

[21] Snelson FF, Rasmussen LEL, Johnson MR, Hess DL (1997) Serum Concentrations of Steroid Hormones during Reproduction in the Atlantic Stingray, *Dasyatis sabina* . General and Comparative Endocrinology 108: 67–79. 9378275 10.1006/gcen.1997.6949

[22] Rasmussen LEL, Gruber SH (1993) Serum concentrations of reproductively-related circulating steroid hormones in the free-ranging lemon shark, *Negaprion brevirostris* . Environmental Biology Fishes 38: 167–174.

[23] Callard IP, Fileti LA, Perez LE, Sorbera LA, Giannoukos G, et al (1992) Role of corpus luteum and progesterone in the evolution of vertebrate viviparity. Amer Zool 32: 264–275.

[24] Rasmussen L, Murru F (1992) Long-term studies of serum concentrations of reproductively related steriod hormones in individual captive carcharhinids. Australian Journal of Marine and Freshwater Research 43: 273–281.

[25] Rasmussen LEL, Gruber SH (1990) Serum levels of circulating steroid hormones in free-ranging carcharhinoid sharks. In: Pratt HL, Taniuchi JT, Gruber SH, editors. NOAA Technical Report NMFS 90 Elasmobranchs as living resources: Advances in the biology, ecology, systematics and the status of the fisheries: U.S. Department of Commerce.pp. 143–155.

[26] Lopes FL, Desmarais JA, Murphy BD (2004) Embryonic diapause and its regulation. Reproduction 128: 669–678. 15579584 10.1530/rep.1.00444

[27] Ewert MA (2004) Cold torpor, diapause, delayed hatching and aestivation in reptiles and birds. In: Deeming DC, Ferguson MWJ, editors. Egg incubation: its effects on embryonic development in birds and reptiles. Melbourne: Cambridge University Press. pp. 173–191.

[28] Atkins N, Swain R, Wapstra E, Jones SM (2007) Late stage deferral of parturition in the viviparous lizard *Niveoscincus ocellatus* (Gray 1845): implications for offspring quality and survival. Biological Journal of the Linnean Society 90: 735–746.

[29] Shanbhag BA, Radder RS, Saidapur SK (2001) Plasma progesterone levels and luteal activity during gestation and prolonged egg retention in a tropical lizard, *Calotes versicolor*. General and Comparative Endocrinology: 73–79. 10.1006/gcen.2001.7647 11551119

[30] Curlewis J (1992) Seasonal prolactin secretion and its role in seasonal reproduction: a review. Reproduction, Fertility and Development 4: 1–23. 10.1071/rd9920001 1585003

[31] Callard IP, Klosterman LL, Sorbera LA, Fileti LA, Reese JC (1989) Endocrine regulation of reproduction in elasmobranchs: Archetype for terrestrial vertebrates. Journal of Experimental Zoology 252: 12–22.

[32] Foresman KR, Mead RA (1978) Luteal Control of Nidation in the Ferret (*Mustela putorius*). Biology of reproduction 18: 490–496. 667254 10.1095/biolreprod18.3.490

[33] Murphy BD, Mead RA, McKibbin PE (1983) Luteal contribution to the termination of preimplantation delay in mink. Biology of reproduction 28: 497–503. 6838952 10.1095/biolreprod28.2.497

[34] Dey SK, Lim H, Das SK, Reese J, Paria BC, et al (2004) Molecular cues to implantation. Endocrine Reviews 25: 341–373. 15180948 10.1210/er.2003-0020

[35] Last PR, Stevens JD (2009) Sharks and rays of Australia. Collingwood: CSIRO Publishing. 513 p.

[36] AIMS (2012) Australian Institute of Marine Science. Data obtained on 23th February 2012 from Online data archives, AIMS. Sea temperatures and marine weather.

[37] USNO (2011) United States Naval Observatory. Astronomical Applications Department Data obtained on 7th April 2011 from Data services, Sun and moon rise/set table for one year. Washington, DC, USA

[38] Nicol S, Andersen NA, Jones SM (2005) Seasonal variations in reproductive hormones in free-ranging echidnas (*Tachyglossus aculeatus*): Interaction between reproduction and hibernation. General and Comparative Endocrinology: 204–210. 10.1016/j.ygcen.2005.05.013 16054627

[39] R Development Core Team (2012) R: A language and environment for statistical computing. R Foundation for Statistical Computing, Vienna, Austria. ISBN 3-900051-07-0, URLhttp://www.R-project.org/.

[40] Savicky P (2009) pspearman: Spearman's rank correlation test. R package version 0.2-5. http://CRAN.R-project.org/package=pspearman.

[41] Koob TJ, Callard IP (1999) Reproductive endocrinology of female elasmobranchs: Lessons from the little skate (*Raja erinacea*) and spiny dogfish (*Squalus acanthias*). Journal of Experimental Zoology 284: 557–574. 10469994 10.1002/(sici)1097-010x(19991001)284:5<557::aid-jez12>3.3.co;2-g

[42] Koob TJ, Tsang P, Callard IP (1986) Plasma estradiol, testosterone, and progesterone levels during the ovulatory cycle of the skate (*Raja erinacea*). Biology of Reproduction 35: 267–275. 3768454 10.1095/biolreprod35.2.267

[43] Craik JCA (1978) An annual cycle of vitellogenesis in the elasmobranch *Scyliorhinus canicula* . Journal of the Marine Biological Association of the United Kingdom 58: 719–726.

[44] Lucifora LO, Menni RC, Escalante AH (2002) Reproductive ecology and abundance of the sand tiger shark, *Carcharias taurus*, from the southwestern Atlantic. ICES Journal of Marine Science: Journal du Conseil 59: 553–561.

[45] Carrier JC, Pratt HL, Castro JI (2004) Reproductive Biology of Elasmobranch. In: Carrier JC, Musick JA, Heithaus MR, editors.Biology of sharks and their relatives.Boca Raton: CRC Press. pp. 269–286.

[46] Castro JI (1993) The biology of the finetooth shark, *Carcharhinus isodon* . Environmental Biology of Fishes 36: 219–232.

[47] Cliff G, Dudley SFJ, Davis B (1988) Sharks caught in the protective gill nets off Natal, South Africa. 1. The sandbar shark *Carcharhinus plumbeus* (Nardo). South African Journal of Marine Science 7: 255–265.

[48] Desmarais JlA, Bordignon V, Lopes FL, Smith LC, Murphy BD (2004) The Escape of the Mink Embryo from Obligate Diapause. Biology of reproduction 70: 662–670. 14585805 10.1095/biolreprod.103.023572

[49] Tsang PCW, Callard IP (1987) Luteal progesterone production and regulation in the viviparous dogfish, *Squalus acanthias* . Journal of Experimental Zoology 241: 377–382.

[50] Sorbera LA, Callard IP (1995) Myometrium of the spiny dogfish *Squalus acanthias*: peptide and steroid regulation. American Journal of Physiology - Regulatory, Integrative and Comparative Physiology 269: R389–R397. 10.1152/ajpregu.1995.269.2.R389 7653661

[51] Ptak GE, Tacconi E, Czernik M, Toschi P, Modlinski JA, et al (2012) Embryonic Diapause Is Conserved across Mammals. PLoS ONE 7: e33027. 22427933 10.1371/journal.pone.0033027 PMC3299720

[52] Lutton BV, St.George J, Murrin CR, Fileti LA, Callard IP (2005) The Elasmobranch ovary. Reproductive biology and phylogeny of chondrichthyes: sharks, batoids and chimaeras. Enfield: Science Publishers, INC. pp. 562.

[53] Lance V, Callard IP (1969) A histochemical study of ovarian function in the ovoviviparous elasmobranch, *Squalus acanthias* . General and Comparative Endocrinology 13: 255–267. 4397545 10.1016/0016-6480(69)90247-0

[54] Chieffi G (1962) Integration of reproductive functions II. Endocrine aspects of reproduction in elasmobranch fishes. General and comparative Endocrinology 1 Supplement 1 275–285. 13878936 10.1016/0016-6480(62)90098-9

[55] Tewinkel LE (1972) Histological and histochemical studies of post-ovulatory and pre-ovulatory atretic follicles in *Mustelus canis* . Journal of Morphology 136: 433–457. 4111712 10.1002/jmor.1051360404

[56] Girling JE, Jones SM (2003) In vitro progesterone production by maternal and embryonic tissues during gestation in the southern snow skink (*Niveoscincus microlepidotus*). General and comparative Endocrinology 133: 100–108. 12899851 10.1016/s0016-6480(03)00147-3

[57] Guarino FM, Paulesu L, Cardone A, Bellini L, Ghiara G, et al (1998) Endocrine activity of the corpus luteum and placenta during pregnancy in *Chalcides chalcides* (Reptilia, Squamata). General and Comparative Endocrinology 111: 261–270. 9707472 10.1006/gcen.1998.7098

[58] Simpfendorfer CA (1993) The biology of the family Carcharhinidae from the nearshore waters of Cleveland Bay, with particular reference to *Rhizoprionodon taylori*. Townsville: James Cook University of North Queensland. 286 p.

[59] Callard IP, Fileti LA, Koob TJ (1993) Ovarian steroid synthesis and the hormonal control of the elasmobranch reproductive tract. Environmental Biology of Fishes 38: 175–185.

[60] Tsang PCW, Callard IP (1987) Morphological and endocrine correlates of the reproductive cycle of the aplacental viviparous dogfish, *Squalus acanthias* . General and Comparative Endocrinology 66: 182–189. 3582950 10.1016/0016-6480(87)90266-8

[61] Sumpter JP, Dodd JM (1979) The annual reproductive cycle of the female lesser spotted dogfish, *Scyliorhinus canicula* L., and its endocrine control. Journal of Fish Biology 15: 687–695.

[62] Awruch CA, Pankhurst NW, Frusher SD, Stevens JD (2008) Endocrine and morphological correlates of reproduction in the draughtboard shrak *Cephaloscyllium laticeps* (Elasmobranchii: Scyliorhinidae). Journal of Experimental Zoology 309A: 184–197. 10.1002/jez.445 18278802

[63] Heupel MR, Whittier JM, Bennett MB (1999) Plasma steroid hormone profiles and reproductive biology of the epaulette shark, *Hemiscyllium ocellatum* . Journal of Experimental Zoology 284: 586–594. 10469996

[64] Mull CG, Lowe CG, Young KA (2008) Photoperiod and water temperature regulation of seasonal reproduction in male round stingrays (*Urobatis halleri*). Comparative Biochemistry and Physiology Part A: Molecular & Integrative Physiology 151: 717–725. 10.1016/j.cbpa.2008.08.029 18793743

[65] Mull CG, Lowe CG, Young KA (2010) Seasonal reproduction of female round stingrays (*Urobatis halleri*): Steroid hormone profiles and assessing reproductive state. General and Comparative Endocrinology 166: 379–387. 20015450 10.1016/j.ygcen.2009.12.009

